# Peptide-Based Low Molecular Weight Photosensitive Supramolecular Gelators

**DOI:** 10.3390/gels8090533

**Published:** 2022-08-25

**Authors:** Bapan Pramanik, Sahnawaz Ahmed

**Affiliations:** 1 Department of Chemistry, Ben-Gurion University of the Negev, Be’er Sheva 84105, Israel; 2 Department of Medicinal Chemistry, National Institute of Pharmaceutical Education and Research Kolkata, Kolkata 700054, India

**Keywords:** peptide, stimuli responsive, gel, trans-cis isomerization, azobenzene, arylazopyrozoles, spiropyran

## Abstract

Over the last couple of decades, stimuli-responsive supramolecular gels comprising synthetic short peptides as building blocks have been explored for various biological and material applications. Though a wide range of stimuli has been tested depending on the structure of the peptides, light as a stimulus has attracted extensive attention due to its non-invasive, non-contaminant, and remotely controllable nature, precise spatial and temporal resolution, and wavelength tunability. The integration of molecular photo-switch and low-molecular-weight synthetic peptides may thus provide access to supramolecular self-assembled systems, notably supramolecular gels, which may be used to create dynamic, light-responsive “smart” materials with a variety of structures and functions. This short review summarizes the recent advancement in the area of light-sensitive peptide gelation. At first, a glimpse of commonly used molecular photo-switches is given, followed by a detailed description of their incorporation into peptide sequences to design light-responsive peptide gels and the mechanism of their action. Finally, the challenges and future perspectives for developing next-generation photo-responsive gels and materials are outlined.

## 1. Introduction

Supramolecular self-assembly, governed by multiple non-covalent interactions, has been explored as a powerful and elegant strategy for the hierarchical bottom-up synthesis of soft materials across length scales [1,2,3,4,5]. Though the individual supramolecular interactions are weak, the resultant interaction is strong enough to make soft materials with different nanostructures, functions, and elegant properties when they work in tandem. An extreme case of higher order self-assembly is the formation of supramolecular gels, basically, semi-solid materials composed of three-dimensional (3D) networked structures with a large amount of entrapped solvents (water in the case of hydrogels and other solvents for organogels) [6,7,8,9,10,11,12,13]. Due to the reversible nature of the supramolecular interactions, such as hydrogen bonding, π−π stacking, hydrophobic interactions, van der Waals interactions, charge-transfer interactions, etc., the resultant gels are highly sensitive to different external stimuli and thus making those gels highly dynamic in nature [14,15,16]. Over the past couple of decades, a plethora of supramolecular gels with structural sophistication and functional variations, particularly aromatic peptides because of their built π-interactions environment, have been reported [17,18,19,20,21].

In light of this, peptides, because of their unique properties, are proven to be an excellent class of building blocks for devising supramolecular gels [22,23,24,25]. They offer a wide range of structural diversity, self-assembling propensities, and morphological variations due to large possible combinations of amino acids which form peptide sequences [26,27,28,29,30,31,32]. In addition, the design rules for the self-assembly of peptides are well documented. Moreover, peptides offer bioactive functionalities, biocompatibility [33,34], and biodegradability [35]. In addition to this, they can possess unique specific functions like cell targeting and environmental responsiveness owing to their bio-active nature [36]. Chemically, the side chains, free amino (–NH_2_), and carboxyl (–COOH) terminus further open up ample opportunities to integrate drugs [37,38], carriers [39,40], and other functional molecules of interest. Due to the chiral nature of the amino acids (except Glycine (Gly)), often, molecular chirality gets transferred to the supramolecular level causing nano-structures with specific chirality [41,42,43]. Additionally, peptides are synthetically accessible due to the well-established straightforward, efficient procedure of Solid Phase Peptide Synthesis (SPPS) that makes them a promising candidate for assembling, programming, and recognizing with utmost efficacy and minimum toxicity [44,45]. Additionally, peptides are well-known for their smaller size (length ranging from 10 to 15 amino acids), even smaller than antibodies, and they are less immunogenic and highly stable in physiological conditions, making them a reliable candidate for conjugation with various kinds of nano-carriers for biological application [46]. Finally, peptides are well-known for their co-assembling and co-aggregating propensity with a wide range of molecular entities such as other peptide sequences, proteins, polymers, drug molecules, inorganic and other molecules [47,48,49,50,51,52,53,54,55]. Co-assembly can occur at the molecular level in mainly four different ways, viz. (a) cooperative co-assembly, (b) self-sorting (or orthogonal co-assembly), (c) random co-assembly, and (d) destructive co-assembly [50]. These newly generated multicomponent co-assembled systems give access to tailored features, enhanced mechanical and architectural scope, desired morphology, improved bioavailability, and functional complexity with emergent behavior [48,49,50,51,52,53,54,55,56,57,58,59]. In fact, in recent years, a considerable amount of effort have been dedicated in the direction of designing peptide-based multicomponent systems decorated with desired structures, properties, and functions with multitasking abilityies via co-assembly, which is difficult for a monocomponent peptide assembly to realize [48,57,58,59,60,61].

One potentially helpful feature of supramolecular gels is their switchable behavior in different physical states in response to various external stimuli. Although a plethora of incentives, for example, ionic strength [62,63,64], pH [44,64], enzyme [65,66,67,68,69], temperature [14,70,71,72], mechanical stress [73], light [74,75], etc., have been reported extensively to show the switching ability, among them, light has received extensive attention because of its non-invasive nature and more importantly, light permits to target a specific area of gel remotely by using photo masks with a high level of spatiotemporal resolution causing patterned gel surfaces and rapid phase transitions reversibly [17,18,76,77,78]. On top of that, the system is free from waste generation/chemical contaminants hence closed systems can be stimulated without introducing any foreign chemicals, and finally, the light can be conveniently switched on and off with specific wavelengths and tunable intensities to modulate and program supramolecular gelation [18,77,79]. Considering the utmost advantages of peptides and light, in recent years, a variety of photo-responsive moieties has been incorporated into the peptides to design photo-responsive gelators which can display switchable, smart, and emergent features [15,80,81,82,83,84].

**Scheme 1:**
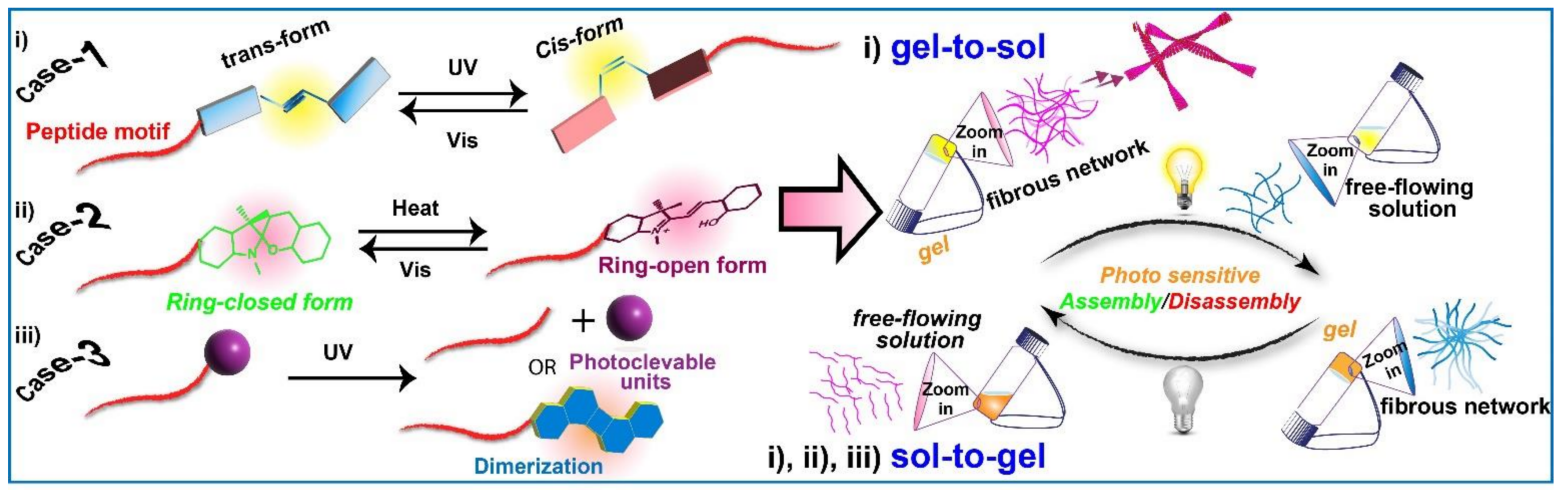
Schematic representation of light-responsive supramolecular gels.

This short review features the recent advancement toward developing low molecular weight supramolecular light-responsive peptide gels. Although a massive number of light-responsive peptide assemblies have been documented in recent years, considering the scope of this short review, we have only included the special cases where ‘gels’ are involved, as shown in Scheme 1.

## 2. Light-Responsive Molecular Switches

**Figure 1:**
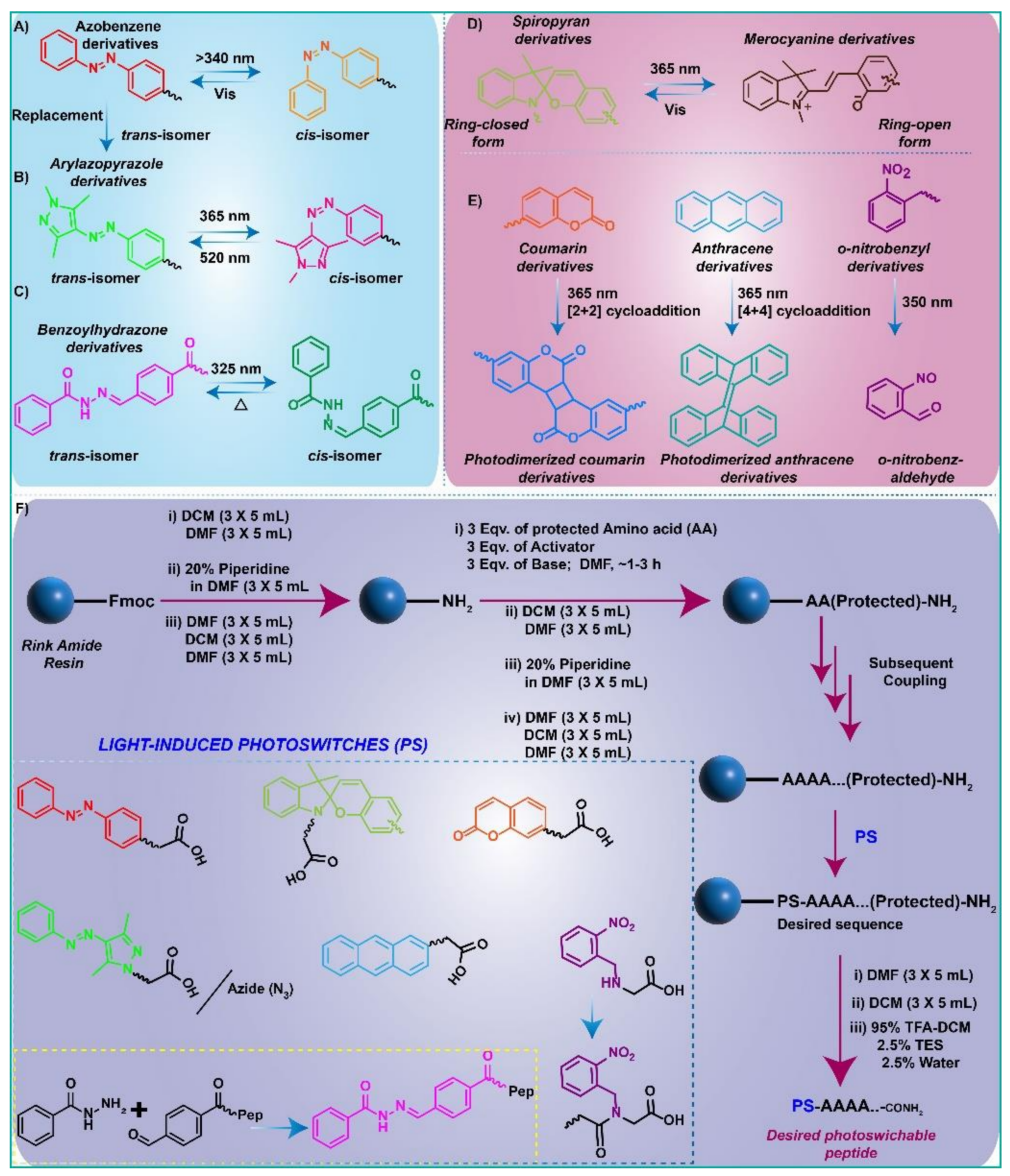
Molecular structure of: (**A**) Azobenzene; (**B**) Arylazopyrazoles; (**C**) Benzoylhydrazone derivatives and light-induced reversible *trans*- and *cis*- isomerization; (**D**) Chemical structure and light-assisted reversible equilibrium between Spiropyran and Merocyanine derivatives; (**F**) Schematic illustration for synthesis of photo-responsive peptide conjugates using SPPS.

In view of light-responsive supramolecular gels, azobenzene [55,85,86,87,88], arylazopyrozole [89,90,91], benzoylhydrozone [92,93], stilbene [60,94], etc., are more frequently used photoisomerizable molecules that switch between *trans*- and *cis*-isomeric forms under the illumination of light (Figure 1). Spiropyran [24,78,80] is another critical photo-sensitive unit for light-induced ring-opening and closing behavior. The 2-nitrobenzyl (NB) group [95,96,97,98], coumarin [99,100,101], anthracene [102,103,104], and diarylethene [105,106] units are also used to create light-responsive gels (Figure 1). The molecular structure and light-induced structural changes of the most well-studied and explored photo-switches in recent years are shown in Figure 1. Additionally, in view of chemical approaches, a schematic illustration is depicted to synthesize the photo-switchable peptide monomers (Figure 1F).

### 2.1. Azobenzene Conjugated Peptide Derivatives and Light-Assisted Self-Assembly/Disassembly Phenomenon

Azobenzene core is the most common photo-responsive moiety incorporated in peptide sequences to design low molecular weight peptide gelators to develop numerous functional soft materials [78,107,108,109,110,111,112,113]. Under UV-light irradiation, the azobenzene core undergoes *trans*- (*E*-) to *cis*- (*Z*-) isomerization, while the reverse *cis* to *trans* isomerization process is carried out by visible light or thermally in a dark environment (Figure 1A) [78,107,108,109,110,111,112,113]. The *trans*-isomer is a thermodynamically favored state. The photoisomerization leads to the change in molecular planarity, which in turn affects π−π stacking interaction amongst the azobenzene moieties causing alteration of the molecular packing of azobenzene-incorporated peptides, which ultimately results in the formation or disruption of gels [109,113,114]. Hence, it is fascinating to incorporate azobenzene into short peptide sequences to create light-responsive peptide hydrogels with variable properties and functions due to light-induced changes in the steric profile of the installed azobenzene.

**Figure 2:**
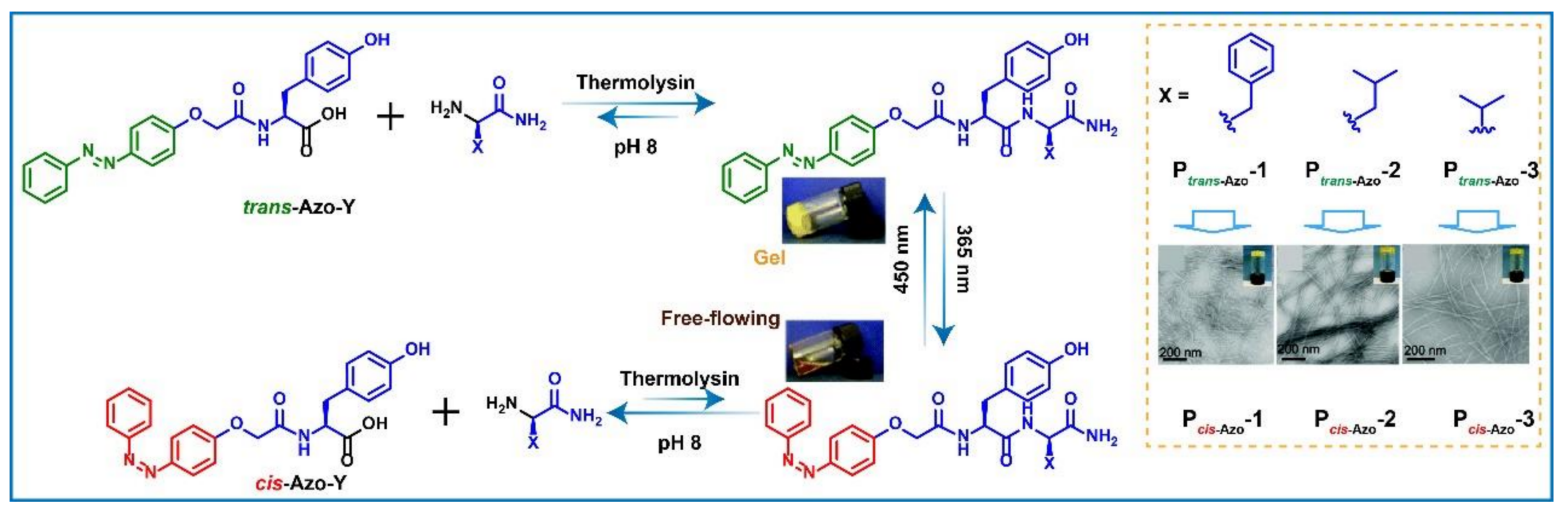
Molecular structure of the synthesized peptides (P*_trans_*_-Azo_−1, P*_trans_*_-Azo_−2, and P*_trans_*_-Azo_−3). Light-induced gel-sol-gel transition (When X = side chain of F) and TEM images of P*_trans_*_-Azo_-1, P*_trans_*_-Azo_−2, and P*_trans_*_-Azo_−3. Adapted from Ref. [112]. Copyright 2014 Royal Society of Chemistry.

Following the light responsiveness of azobenzene, Prof. Rein Ulijn and coworkers demonstrated the integration of light switching with enzymatic amide formation/hydrolysis to form and manipulate low-molecular-weight peptide gelation [112]. In this work, they started with a non-gelator molecule *trans*-Azo-Y (Y for tyrosine, Figure 2) and synthesized a series of peptides, P*_trans_*_-__Azo_−1, P*_trans_*_-Azo_−2, and P*_trans_*_-Azo_−3, using thermolysin catalyzed amidation with X (X represents the side chain of phenylalanine (F), leucine (L) and valine (V)). These dipeptides exhibit gelation at different time intervals after adding the enzyme thermolysin. The contributing interactions of the gelation can be accredited to π−π stacking between *trans*-azobenzene moieties and aromatic amino acids, combined with hydrogen bonding among dipeptide units. Rheological analysis revealed that the storage modulus (G′) of the dipeptide hydrogels (10^4^–10^5^ Pa) is higher than their corresponding loss modulus (G″, 10^3^–10^4^ Pa), suggesting the gel property of the hydrogels (Table 1). Among the dipeptides, P*_trans_*_-Azo_−1 gel was tested for displaying light responsiveness, and when it was exposed to a UV lamp (365 nm), the hydrogel disintegrated and dissolved after 48–72 h of illumination (Figure 2). This gel to sol transition of P*_trans_*_-Azo_−1 was observed due to the conformational switching of the azobenzene from planar *trans*-(*E*) to non-planar *cis*-(*Z*) form. The *cis*-isomer prohibits adequate π−π stacking and hydrophobic interaction between azobenzene moieties required for gelation. On further exposure to visible light (450 nm), the reverse isomerization, i.e., *cis*- to *trans*-form, restored gelation due to reinstatement of the favorable supramolecular interactions. The authors claimed that this light-induced trans-cis isomerization also results in a condition where the thermolysin catalyzed hydrolysis favors condensation. They validated this behavior by comparing the high-performance liquid chromatography (HPLC) yields of the bio-catalytic condensation reaction of amidated F with *trans*-Azo-Y and *cis*-Azo-Y.

**Table 1:** Parameters of the photo-switchable peptide gels and photo-responsive behavior.

SL. No	System	Minimum gelation conc. (MGC, wt% and mg mL^−1^)	Media	Light	Moduli (Before Light irradiation) Pa		Moduli (After UV-Light irradiation) Pa		Moduli (After Vis-Light irradiation) Pa		Morphology
					G′	G″	G′	G″	G′	G″	
***Azobenzene (Azo) derivatives***											
1.	**P*_trans_*_-Azo_−1** [112]	17 mM	Phosphate buffer (pH 8.0)	365 nm (UV) 450 nm (Vis)	~10^4^–10^5^	~10^3^–10^4^	NR	NR	NR	NR	Nanofibers Micellar aggregates (UV) Nanofibers (Vis)
2.	**OGAC/ AzoC_2_Py (5:1)** [115]	0.13 wt% of OGAC and accordingly AzoC_2_Py	Water	365 nm (UV) >400 nm (Vis)	NR	NR	NR	NR	NR	NR	Long nanofibers Helical nanofibers (UV) Thick and straight Fibers (Vis)
3.	**P*_trans_*_-Azo_−6** [74]	16.2 mM	Water (pH~10)	365 nm (UV)	~10^2^	~10	~10^3^	~10^2^	NA	NA	Thin fibers Long rod (UV)
4.	**P*_trans_*_-Azo_−7** [87]	15 mg mL^−1^	PBS (pH 7.4)	530 nm (GL) 410 nm (Vis)	~10^4^	~10^3^	NR	NR	NR	NR	Fine fibers
***Arylazopyrazoles (AAP) derivatives***											
5.	**P-1+ P_AAP_−1+CDV** [116]	2.5 wt% of P-1 and 0.25 wt% of P_AAP_−1	Water	365 nm (UV) 520 nm (Vis)	~10^5^	10^4^–10^5^	10^2^–10^3^	~10^2^	10^4^–10^5^	~10^4^	Cross-linked fibers
6.	**P-2+ P_AAP_−2+CDVs** [117]	1.0 wt% of P-2 and 20 % of P_AAP_−2	Water	365 nm (UV) 520 nm (Vis)	~27 × 10^3^	NR	~24 × 10^3^	NR	~26.5 × 10^3^	NR	NR
7	**P_AAP_−3** [118]	5.0 wt%	Water	365 nm (UV) 520 nm (Vis)	~7.8 × 10^3^	~10	~6.3 × 10^3^	>1	~8 × 10^3^	~100	NR
8.	**P_AAP_−3 +Agarose** [118]	5.0 wt% of P_AAP_−3 and 1.7 wt % of Agarose	Water	365 nm (UV) 520 nm (Vis)	~6.8 × 10^3^	~1	~6.3 × 10^3^	>1	~8.3 × 10^3^	~100	NR
9.	**P_AAP−_4** [90]	2 mg mL^−1^	Water (pH~5)	365 nm (UV) 520 nm (Vis)	325–350	~25	125–225	~50	150–300	~50	Aggregate
10.	**P_AAP_−5** [90]	2 mg mL^−1^	Water (pH~5)	365 nm (UV) 520 nm (Vis)	140–180	<25	140–170	~25	200–300	50–75	Cross-linked fibers
***Spiropyran (SP) derivatives***											
11.	**P_MC_−4** [119]	11 mM	Water (pH 3)	254 nm (UV) 420 nm (Vis)	NR	NR	NR	NR	NR	NR	Fibers
12.	**SPI-RGD** [120]	4.0 mg mL^−1^	Water (pH 5.2)	365 nm (UV) 420 nm (Vis)/∆	300	~80	2.3	~NR	~315–335	NR	Fibers
13.	**P_SP−5_** [121]	10 mM	Water	254 nm (UV) 420 nm (Vis)/∆	NA	NA	NR	NR	NA	NA	No discernible structures Twisted nanofibrils (Vis)
***Coumarin (Cou) and anthracene (Anth) derivatives***											
14.	**P_Cou_−1** [122]	10mM	365 nm	Water (pH 7)	~20 (Pa)	~10 (Pa)	~150 (Pa)	~70 (Pa)	NA	NA	Fibers
15.	**P_Cou_−2** [22]	5 mg mL	365 nm	Water (pH)	10^3^–10^4^	10^2^–10^3^	~10^4^	~10^3^	NA	NA	Fibers
16.	**P_Cou_−3** [123]	2.7 mg mL^−1^	365 nm	PEG200:H_2_O = 1:2.	NR	NR	NA	NA	NA	NA	Spiral-shaped fibers Fibers (UV)
17.	**P_Anth_−1** [102]	5 mg mL^−1^	350 nm (UV)	water	10^2^–10^3^	10^1^–10^2^	0.1–10	0.01–1.0	NA	NA	Nanoribbons
***Other derivatives (*Benzoylhydrazone *(*BHz*), Nitrobenzyl (NB) and 6-nitroveratryloxycarbonyl (Nvoc))***											
18.	**BHz-F(F)(F)** [92]	0.5 wt%	325 nm	MES buffer (pH 7.0)	~10^4^	~10^3^	NR	NR	10^3^–10^4^	~10^3^	Nanofibers NR (UV) Nanofibers (Dark)
19.	**MAX7CNB** [124]	2 wt%	(260 < λ < 360 nm)	Water (pH 9)	10^3^–10^4^	~10^2^	~10^3^	~10^2^	NA	NA	Fibers
20.	**P_NB_−1** [125]	4.0 × 10^3^ M	350 nm (UV)	Water	NA	NA	~10^5^	~10^4^	NA	NA	Quadruple helix (Before UV) Cylindrical fibrils
21.	**P_NB_−2** [126]	4.0 × 10^3^ M	350 nm (UV)	Water	NA	NA	~10^5^	~10^4^	NA	NA	Spheres (Before UV) Fibers
22.	**P_NB_−3** [127]	1.70 mM	350 nm (UV)	Water (pH 7.4)	~1–2	~1	NA	NA	NA	NA	Long and tangled fibers Less and finer fibers (UV)
23.	**NVOC-FF** [128]	5 mg mL^−1^	365 nm (UV)	DMSO:Water (5:95)	10^3^–10^4^	10^2^–10^3^	~1	NR	NA	NA	Fibers

N.B: G′: Storage modulus; G″: Loss modulus; NR: Not reported; NA: Not applicable; GL (Green Light); ∆: heat.

**Figure 3:**
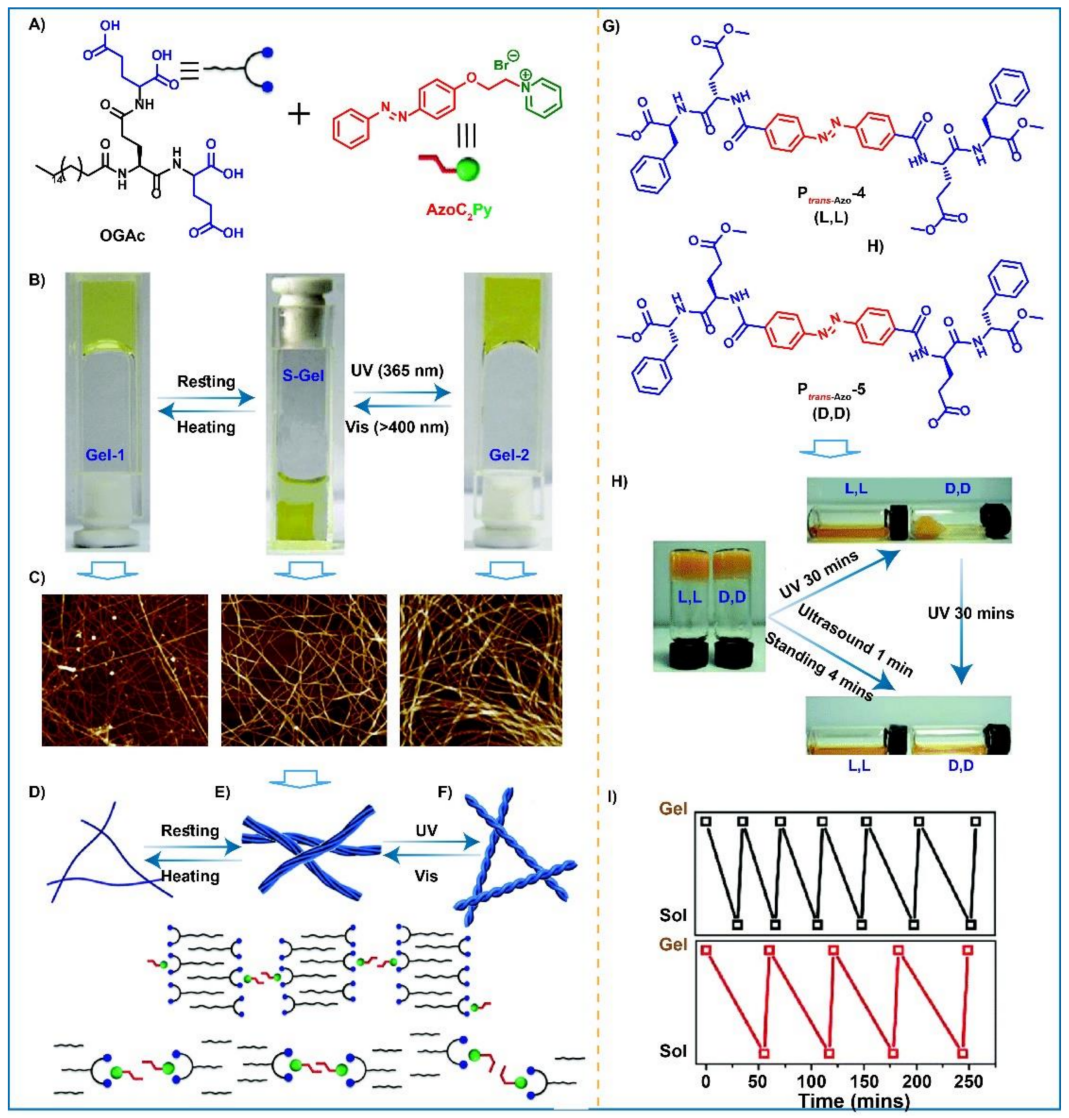
(**A**) Chemical structure of the precursors; (**B**) Stimuli-responsive gel-sol-gel transition; (**C**) AFM images of the physical states mentioned in (**B**); Schematic illustration of hierarchical co-assembly: (**D**) Thin layer fibril formation in fresh hydrogel; (**E**) Thick fibrous bundles in shrunken gel; (**F**) Helices formation in light-induced swollen gel. Adapted from Ref. [115]. Copyright 2016 The Royal Society of Chemistry. (**G**) Molecular structure of the peptide gelators (P*_trans_*_-Azo_−4 and P*_trans_*_-Azo_−5); Redrawn from Ref. [120]. Copyright is not applicable here; (**H**) Light-responsive gel-sol-gel transition of P*_trans_*_-Azo_−4 and P*_trans_*_-Azo_−5; (**I**) Reversibility test for gel-sol-gel transition, black and red curves represent L gel and D gel respectively. Adapted from Ref. [130]. Copyright 2017 The Royal Society of Chemistry.

Minghua Liu and coworkers adopted a co-assembly approach to realize supramolecular dendron gel, which shows shrinking/swelling behavior upon photoirradiation and thermal switch (Figure 3) [115]. In this case, mixing an amphiphilic dendron terminated with three L-glutamic acid groups (OGAc) and a positively charged azobenzene derivative, AzoC_2_Py (Figure 3A), produced Gel-1 (Figure 3B). Gel-1, when kept at 20 °C, shrank to form a shrunken gel (S-gel, Figure 3B) by expelling water molecules due to the aggregation of the hydrophobic *trans*-azobenzene moiety. On exposure to UV light, the *trans*- to *cis*- isomerization occurred, which led to the swelling of the shrunken gel to form Gel-2. The Gel-2 is relatively stable at room temperature unless the gel is subjected to visible light leading to regeneration of S-gel due to *cis*- to *trans*- isomerization, and this swelling between the S-gel and Gel 2 can be reversibly switched by alternate Vis/UV irradiation several cycles (Figure 3B). Moreover, S-Gel to Gel-1 interconversion can be achieved by a thermal switch, thereby making a system dual responsive, exhibiting three gel states. At the microscopic level, Gel-1 exhibited nanofiber morphology, whereas for S-gel, thick and straight, and Gel-2, helically entangled fibrous structures were observed (Figure 3C). Based on different spectroscopic and microscopic observations, the authors proposed a possible mechanism, as shown in Figure 3D–F, where OGAc adopted an interdigitated bilayer structure and AzoC_2_Py occupied the head position of the bilayer leading to the formation of a fibrous structure. Due to its hydrophobic nature and π−π staking ability, the *trans*-azobenzene aggregated over time, and consequently, the water molecules were expelled, causing the shrinking of the gel. The UV light-induced *trans*- to *cis*- isomerization resulted in a volume change of the azobenzene moiety. Consequently, the water molecules were taken into the bilayer, and swelling occurred to form Gel-2. Next year, the same group utilized the aforementioned co-assembly approach to demonstrate a series of photo-responsive gels with the help of alkylated-L-Histidine and carboxylic acid substituted-azobenzenes [129].

In an exciting work, Zhonghui Chen et al. reported a pair of dipeptide appended-azobenzene photo-responsive reversible chiral gelators (P*_trans_*_-Azo_−4 and P*_trans_*_-Azo_−5, Figure 3H), where the chirality plays an essential role in the photo-induced gel-sol transition [130]. The gelators are composed of an azobenzene flanked between L-Asp-L-Phe (P*_trans_*_-Azo_−4) and D-Asp-D-Phe (P*_trans_*_-Azo_−5). The L-gel (i.e., the gel formed from P*_trans_*_-Azo_−4) converted into sol much faster upon UV irradiation than the D-gel (i.e., the gel formed from P*_trans_*_-Azo_−5). The authors claimed that the dipeptide units’ molecular chirality determines the molecules’ orientation and molecular packing. These eventually modulate the photo-induced *trans*- to *cis*- isomerization rates of azobenzene moiety, causing dissimilar disassembly kinetics of the two gels. The gels also exhibited light-induced multiple gel-sol transitions, but at different rates for the L-gel and D-gel systems (Figure 3I).

**Figure 4:**
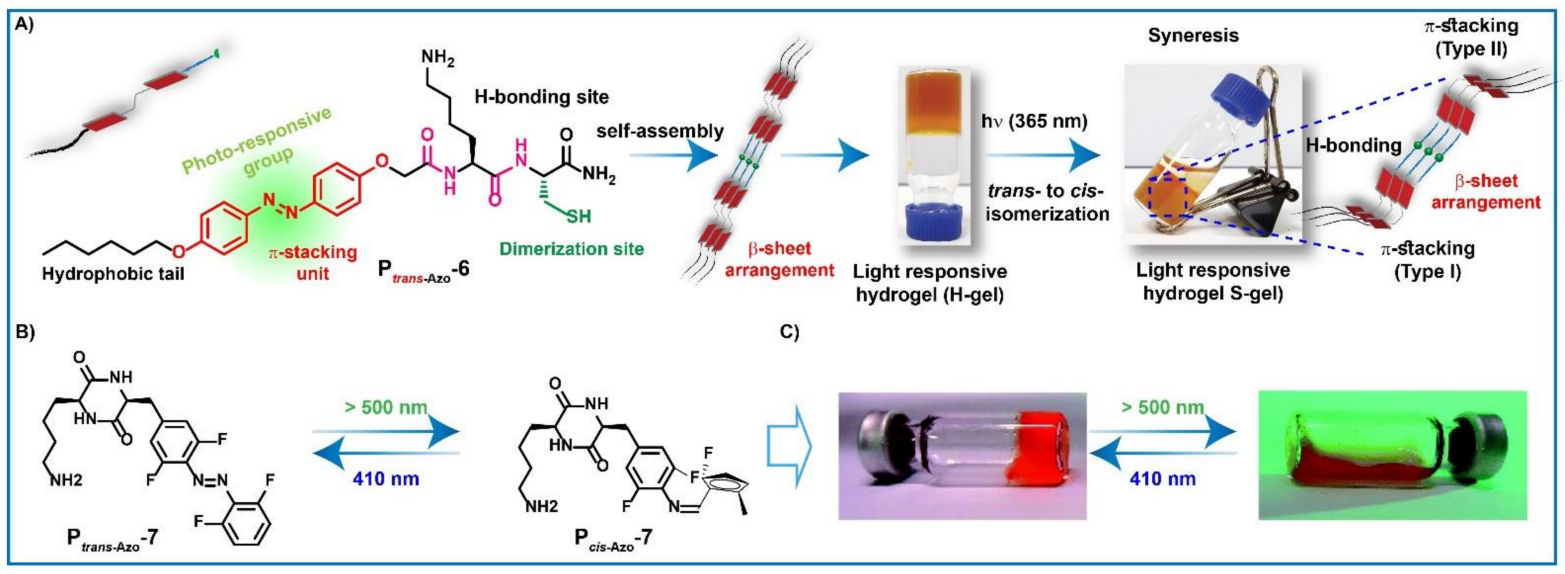
(**A**) Molecular structure, self-assembly mechanism, photographs of formed gel and UV-light induced possible molecular arrangement of the Azo-peptide hydrogelator, P*_trans_*_-Azo_−6. Adapted from Ref. [74]. Copyright 2020 Royal Society of Chemistry. (**B**) Light-induced *trans*- to *cis*- isomerization of the cyclic peptide; (**C**) Photographs of light-sensitive gel-sol transition. Adapted from Ref. [87] Copyright 2021 Royal Society of Chemistry.

In 2020, Das and co-workers showcased a short peptide-based water insoluble and thixotropic hydrogel, which exhibits syneresis and expel water when exposed to UV irradiation (Figure 4A) [74]. In this work, an azobenzene functionalized short peptide, P*_trans_*_-Azo_−6, undergoes self-assembly in fresh aqueous NaOH solution through different non-covalent interactions like π-stacking, H-bonding, hydrophobic, and disulfide bond formation to form a self-supporting hydrogel (H-Gel, Figure 4A). Surprisingly, the formed H- Gel is insoluble in water, and as a result, it restricts the movement of water to and from the gel. When illuminated with UV light (365 nm), the H-Gel displayed an irreversible shrinkage by 50% of its volume by expelling water and formed S-Gel (Figure 4A). There was a shift of morphology from fibers to mixed fibers and rods. Moreover, the S-gel was so strong that neither the Vis light nor the standard disulfide breakers could disrupt the gel due to more robust packing than the H-Gel. The mechanical superiority of the S-Gel over H-Gel was further confirmed by rheological experiments where the G′ value S-Gel and G-Gel lie in the range of ~10^3^ Pa and ~10^2^ Pa, respectively, in the linear viscoelastic region (Table 1). The authors thoroughly investigated the unusual behavior of light-induced syneresis and gel state morphology transfer where the dynamics of the constituent molecules remained highly restricted. It was claimed that during the *trans*- to *cis*- isomerization, the P*_trans_*_-Azo_−6 dimer adopted a new arrangement where the gel requires less water to sustain the assembly and consequently expelled excess water. Finally, the authors exploited this syneresis property of the gel to remove model dye molecules from water. In another report, azobenzene incorporated collagen model peptide hydrogels having light-triggered phase transition behavior was reported by Koga’s group [131].

Recently, Pianowski and co-workers presented a cyclic dipeptide-conjugated azobenzene hydrogelator (P*_trans_*_-Azo_−7) that exhibited photo-induced reversible gel-sol transition (Figure 4B) [87]. In this work, they synthesized a tetra-ortho-fluorinated azobenzene-cyclic dipeptide hydrogelator conjugate considering the fact that aromatic C–F bonds improve supramolecular interactions in the proximity of the fluorine atoms to reduce the minimum gelation concentration (MGC) of the gelator. The gelator forms stable and homogenous hydrogels in aqueous solutions under physiological conditions (PBS buffer, pH 7.4) with an MGC of 17 g/L. The existence of strong hydrogel was confirmed by the rheometric analysis, where the G′ and G″ values were found to be around 10^4^ Pa and 10^3^ Pa, respectively, within the linear viscoelastic region (Table 1). Upon irradiation with green light (530 nm), the hydrogel dissolved and formed a homogeneous solution which, on further treatment with violet light (410 nm) followed by incubation at room temperature in darkness, converted back to the transparent hydrogel again. This phase transition was again due to photo-induced isomerization of the azobenzene moiety. Finally, the authors exploited this hydrogel to encapsulate an anti-cancer drug, plinabulin, for light-induced release without any significant passive diffusion (leaking).

### 2.2. Arylazopyrazoles Conjugated Peptide Derivatives with Light-Sensitive Gelation Characteristics

Although a lot of progress has been made with azobenzene as a light-responsive molecular switch, certain disadvantages restrict their application [132]. For example, the UV-light used to trigger *E*→*Z* isomerization is harmful and can be vastly distributed in biological tissue or nanomaterials [132,133,134]. Additionally, most azobenzene derivatives exhibited low thermodynamic stability of the *Z*-form in comparison with other molecular photo-switches [132]. Consequently, incomplete photoisomerization behavior is noticed owing to the overlapping absorbances of both *E*- and *Z*-isomers. The photostationary state (PSS) for classic azobenzene derivatives is about 80% for the *E*→*Z* and 70% for the *Z*→*E* isomerization [132,135]. Because of this drawback, in highly multivalent systems, a substantial fraction of the remaining *E*-isomer can still dictate the material properties, causing partial switching [132,135,136,137].

For the last few decades, researchers have been trying to develop azobenzene derivatives that can undergo visible light-induced isomerization to aim either to move the π→π* transition to a longer wavelength or to acquire a splitting n→π* transition of the *E*- and *Z*-isomer that typically fuse in 400–500 nm wavelength window [132,138]. Therefore, to solve the issue, the pyrazole hetero cycle was introduced [132,139]. The replacement of one benzene ring in azobenzene with a pyrazole ring resulted in arylazopyrazoles (AAP, Figure 1B), as an alternative and a new light-responsive molecular switch. Introduced by Fuchter et al. they have received enormous attention to the peptide chemist and pharmacist because of their ease and scalable synthesis, good water solubility, and superior photophysical properties [139,140]. As expected, APP displayed a noteworthy red shift of the n→π* transition band of the *Z*-isomer, enabling almost quantitative isomerization by UV(*E*→Z) or green light (*Z*→*E*) irradiation [132]. Additionally, AAP showed half-life times up to 1000 days, which can be attributed to the decreased steric repulsion within the *Z*-form [132].

**Figure 5:**
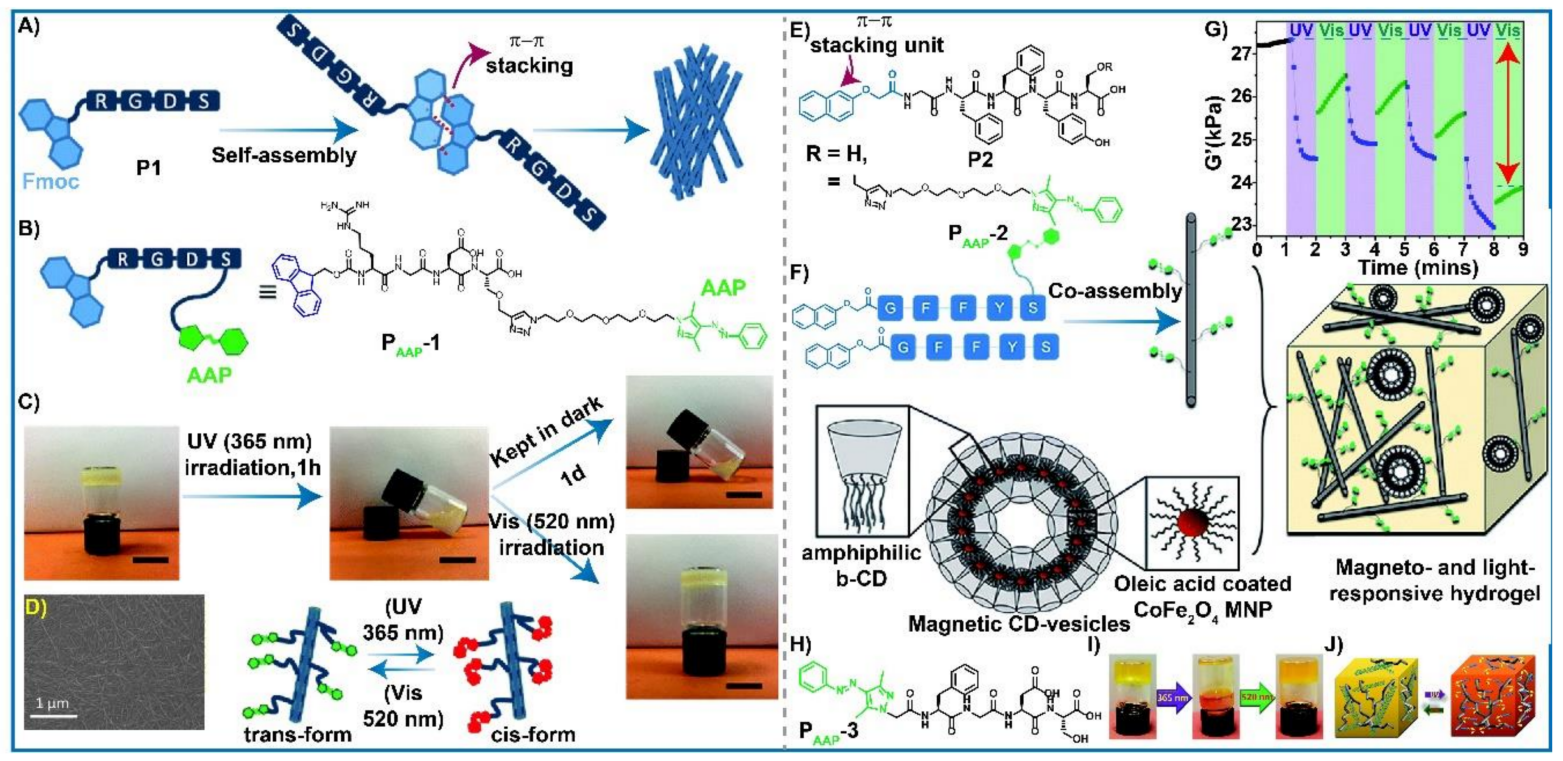
(**A**) Chemical structure and plausible self-assembly mechanism of peptide, P1; (**B**) Molecular structure of AAP modified peptide; (**C**) Gel-sol-gel transition under illumination of UV- and Vis-light respectively; (**D**) Microscopic images of the resulting gel. Adapted from Ref. [116] Copyright 2017 Royal Society of Chemistry. (**E**) Chemical structure of the peptides (without AAP and with AAP) for co-assembly; (**F**) Schematic presentation of the stimuli-responsive hydrogel formation through co-assembly approach; (**G**) Light-responsive rheological behavior of the resultant gel. Adapted from Ref. [117] Copyright 2019 Royal Society of Chemistry. (**H**) Molecular structure of AAP modified peptide; (**I**) Light-triggered gel-sol-gel transition; (**J**) Schematic visualization of the created network. Adapted from Ref. [118] Copyright 2020 Royal Society of Chemistry.

Based on these outstanding properties of AAP, in 2017, Ravoo’s group introduced a unique hierarchical supramolecular hydrogel utilizing both self-assembly and host–guest interaction of the designed amphiphilic peptides [116]. The peptides comprise a tetrapeptide building block, Fmoc-RGDS (P1, Figure 5A), owing to its good water solubility and excellent biocompatibility. The serine side chain was functionalized with AAP through a TEG spacer with the help of click chemistry to design another peptide (P_AAP_−1, Figure 5B) to create a light-responsive stable gel. The gel was fabricated with the combination of P1, P_AAP_-1, and cyclodextrin vesicles (CDV). CDV is a macrocyclic host towards *trans*-AAP as a guest with multivalent non-covalent crosslinking properties. The entanglement of self-assembled supramolecular nanofibers and host-guest interaction between the *trans*-AAP and CDV creates self-supportive hydrogels (Figure 5C, D). UV light irradiation forces *trans*-AAZ to convert its *cis*- form and hence, destroy the host-guest interaction as *cis*-AAP is very reluctant to bind CDV (Figure 5C). Therefore, a very soft non-supporting gel was reached. The reversibility of the hydrogel was achieved either by storing the gel in the dark for four days or by visible light irradiation. This can be ascribed to the non-covalent host–guest interaction, which was restored under experimental conditions. Unfortunately, the reversible gel failed to reach its initial moduli (G′ and G″, Table 1). Next, the same group established another light-responsive hydrogel utilizing a co-assembly approach [117]. The beauty of their finding is that the formed gel is labile towards both light and magnet and, as a result, it showed different rheological behavior (Table 1). The co-assembly between P2 and AAP-modified photo responsive peptide, P_AAP_−2, and superparamagnetic nanoparticles (CoFe_2_O_4_) embedded CDVs creates the distinctive gel (Figure 2E, F). The gel showed around a 10% decrease of the storage modulus (G′) in response to UV light irradiation (350 nm). The gel exhibited a continuous decline in its G′ value compared with the initial value during the reversibility experiment (UV-Vis-UV, Figure 5G) and continued up to four cycles. Next year, Ravoo et al. again presented a hybrid, photo-responsive dual gel network without any external crosslinker made of AAP anchored LMWG, P_AAP_−3, and agarose, a covalent polymer network (Figure 5H) [118]. In response to light, the peptide exhibited reversible gel-to-sol transition with 1 Equiv. of KOH. Although, in the presence of agarose, the storage modulus of P_AAP_−3 displayed two-fold higher magnitude, no signs of P_AAP_−3 leakage out of the non-responsive agarose network was tracked. Surprisingly, in the presence of agarose, there are no macroscopic changes of the formed hybrid gel upon UV and Vis light irradiation, which can be observed from photo responsive rheological experiment also (Table 1). This can be attributed to the formation of the dual network by the precursor gelator components.

**Figure 6:**
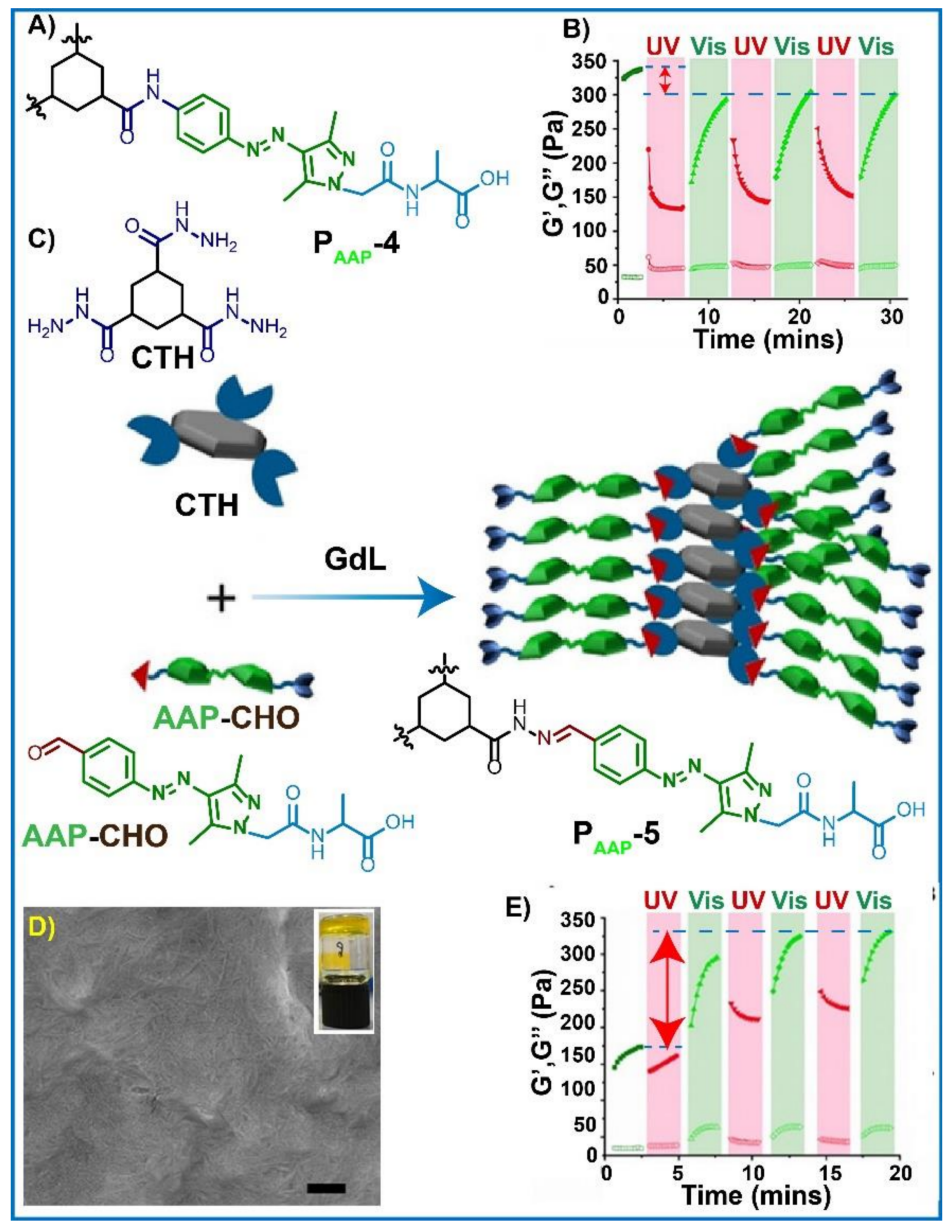
(**A**) Molecular structure of the hydrogelator, P_AAP_−4; (**B**) UV-Vis light-induced rheological experiment; (**C**) In situ gel formation through GdL hydrolysis; (**D**) Microscopic fibrous network of the in-situ gel; (**E**) Light-activated rheological analysis of in-situ hydrogel. Adapted from Ref. [90] Copyright 2019 John Wiley and Sons.

Interestingly, Ravoo and co-workers masterminded a family of tripodal photo-responsive hydrogelators in which cyclohexane-1,3,5-tricarboxamide (CTA) and a cyclohexanetrishydrazide (CTH) act as a central core to provide sufficient π−π stacking and a terminal alanine as an arm to provide water solubility and additional hydrogen bonding (Figure 6) [90]. 

For CTH-based LMWG, an aldehyde terminated AAP peptide (AAP-CHO) undergoes glucono-d-lactone (GdL) induced dynamic hydrazone linkage formation with CTH-hydrazide to form a gel (Figure 6C). At room temperature, G′ was found to be higher than G″, indicating gel characteristics. Under the influence of UV light (λ = 365 nm), the gel (Figure 6E) showed a 50% drop in G′ value, emphasizing the AAP unit’s photoisomerization (Table 1). It is important to highlight that although the gel recovered the G′ value under Vis light irradiation, for reaching a plateau, more time is needed compared with UV irradiation. However, G” value remains constant throughout the experiment. This can be explained by the oscillation and network behavior of the fibers. In contrast to P_AAP_−5 gel, dynamic covalent hydrogel displayed a 30% decrease in G′ value after the first UV irradiation (Figure 6B, Table 1). The photo-responsive rheology experiment showed similar behavior to P_AAP_−4, but the gel becomes stiffer after Vis irradiation, reflecting a higher G′ value (Figure 6E). 

### 2.3. Spiropyran Conjugated Peptide Derivatives and Light-Induced Gelation Behaviour 

**Figure 7:**
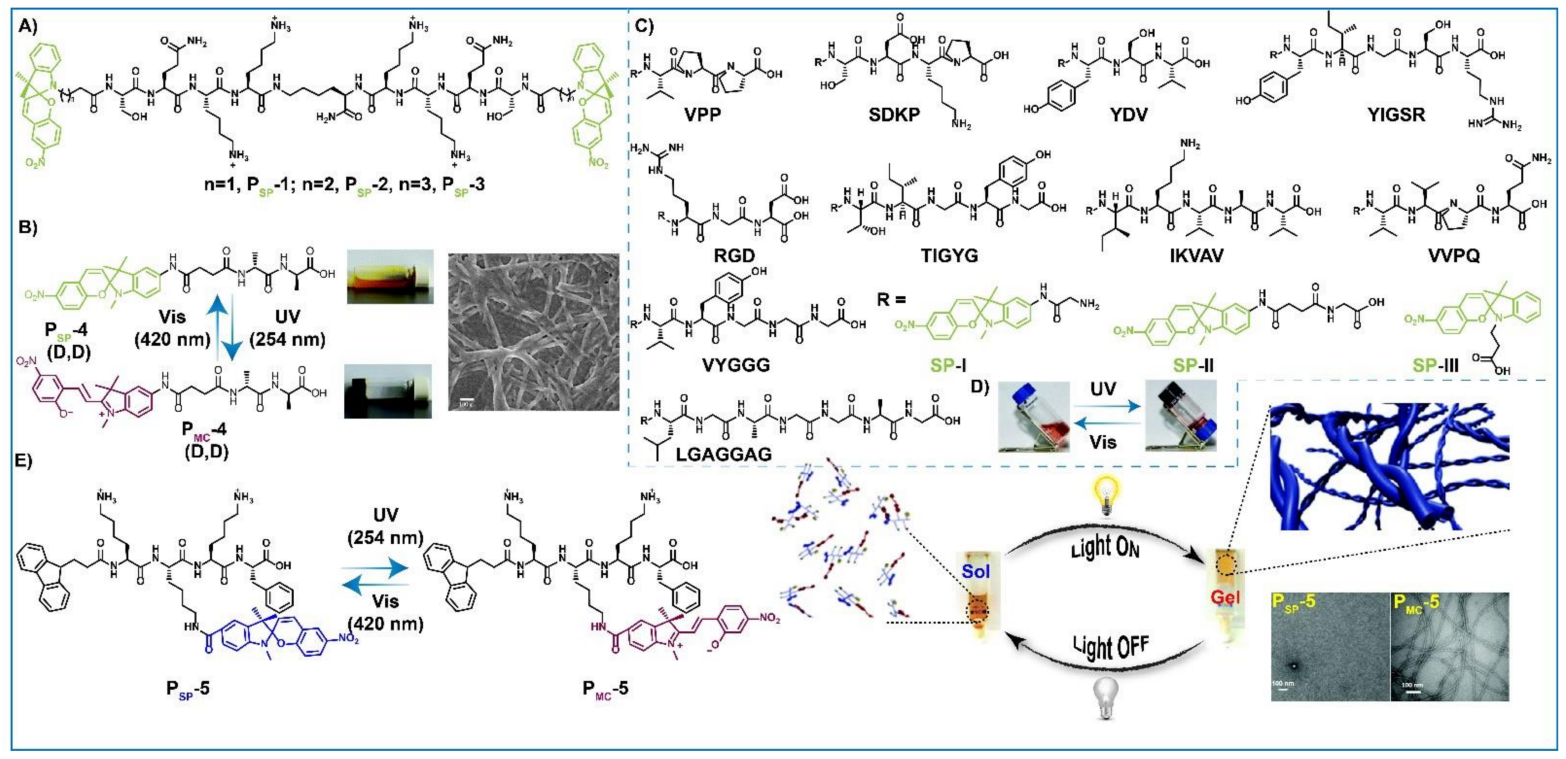
(**A**) Molecular structure of SP-conjugated peptides (P_SP_−1, P_SP_−2, and P_SP_−3); Redrawn from Ref. [153]. Copyright is not applicable here. (**B**) Light-induced sol-gel transformation of SP and MC appended peptides. Adapted from Ref. [119] Copyright 2009 Royal Society of Chemistry (**C**) Chemical structure of the SP analogs and connecting peptide motifs; Redrawn from Ref. [120]. Copyright is not applicable here. (**D**) Photographs of sol and gel because of the light-instructed structural change from SP to MC form. Adapted from Ref. [120] Copyright 2015 The Royal Society of Chemistry. (**E**) Schematic illustration of light-induced dissipative self-assembly of an SP-peptide conjugate. Adapted from Ref. [121] Copyright 2021 The Royal Society of Chemistry.

In light of the molecular photo-switches, spiropyrans have received extraordinary attention from photo chemists and peptide chemists because of their outstanding photophysical properties [141]. Depending on the nature of illuminating light, two distinct structural thermodynamically stable isomers exist with the gigantic difference in properties: (i) colored planar merocyanine (MC) form, a charged hydrophilic ring-open form, and (ii) colorless non-planar spiropyran (SP) form, a non-charged hydrophobic ring-closed form which ultimately make spiropyran a unique photo-switch (Figure 1D) [119,141,142]. Because of the planar structure, SP shows a high propensity to form aggregate-like structures through intermolecular π−π stacking (Figure 1D) [119,121,143]. It is well-documented in literature that a range of stimuli such as temperature, solvents, redox potential, acids, bases, metal ions, mechanical forces, etc., can stimulate spiropyran’s reversible isomerization [144,145,146,147,148,149]. Based on the properties mentioned above, increasing effort has been made to create spiropyran appended novel materials over the decades [150,151,152]. In 2014, Chen and Zhu et al. created a family of antibacterial peptides (P_SP_−1, P_SP_−2, and P_SP_−3, Figure 7A), of which SP units were linked to both ends of the sequence in accordance with varying chain lengths [153]. Under exposure to light, these peptides adopt different thermodynamically stable states (MC and SP) at physiological pH.

In this context, an SP-linked dipeptide (SP-D-Ala-D-Ala, P_SP_−4, Figure 7B) hydrogelator was reported by Zhang et al., which forms hydrogel (pH 3) with a fibrous network in response to light [119]. Under UV light exposure, the non-planar SP form gets converted to planar MC form and undergoes intermolecular π−π stacking to form an aggregated structure. The gel turned into a yellow slurry upon visible light irradiation, owing to disassembly. Later, the same group created a library of peptides (VPP, RGD, YDV, SDKP, VVPQ, YIGSR, TIGYG, IKVAV, VYGGG, and LGAGGAG) conjugated with SPs (SPI, SPII, and SPIII), which form hydrogel at a particular pH based on the connecting sequence (Figure 7C) [120]. Amusingly, the MC form can be achieved at 70 °C with 80% yield within 3 min, and exposure to sunlight for 0.5 min reverts to its SP form (Figure 7D). Remarkably, such amazing heat-light-induced isomerization is completely reversible and can be repetitive for more than five cycles. The rheology cycle also confirms the reversibility of the gel (Table 1). As an application, the authors employed the MCI-RGD gel for an erasable photolithograph material. In 2020, Stupp and Schatz et al. engineered a hybrid photo-responsive soft material to mimic the mechanical actuation [154]. The material was prepared from peptide amphiphile supramolecular polymers covalently anchored with SP. Upon UV irradiation, the formed gel expelled water to shrink to 84% of its original volume, and that is only because of the isomerization phenomenon. Interestingly, the shrunken gel reverts to its original swollen shape when kept in a dark place. Last year, a tetrapeptide (Fmoc-KK(SP)KF-NH_2_, P_SP_−5, Figure 7E) was reported by Parquette in which nitro-SP connected to the ε-amino lysine sidechain of the sequence to provide the light-responsive hydrogelator [121]. The peptide remains in a solution state, but under the illumination of light, the solution readily transforms into a gel with a fibrous network. The gel achieved a free-flowing state when the light was switched off. Interestingly, the cycle can be repeated for multiple cycles. Thus, the system was put under the category of dissipative self-assembly, where the system needs a continuous source of energy in the form of light to sustain. Once the light irradiation is stopped, the system disassembles.

### 2.4. Other Photo-Responsive Peptide Derivatives and Light-Induced Gel-Sol Transition or Vice-Versa 

Coumarins are well-known for their photodimerization tenacity when irradiated with light of wavelength greater than 280 nm (Figure 1E) [22,155]. The photo-induced nature of the coumarins has inspired scientists to prepare stimuli-responsive LMWGs [22,122]. As anticipated, the solubility of the light-induced dimerized coumarin decreases as the coumarin monomer becomes double in size. As a result, hydrophobicity of the system increases, which disrupt the gel network and, eventually, decreases in rheological parameters observed [122,123,156].

**Figure 8:**
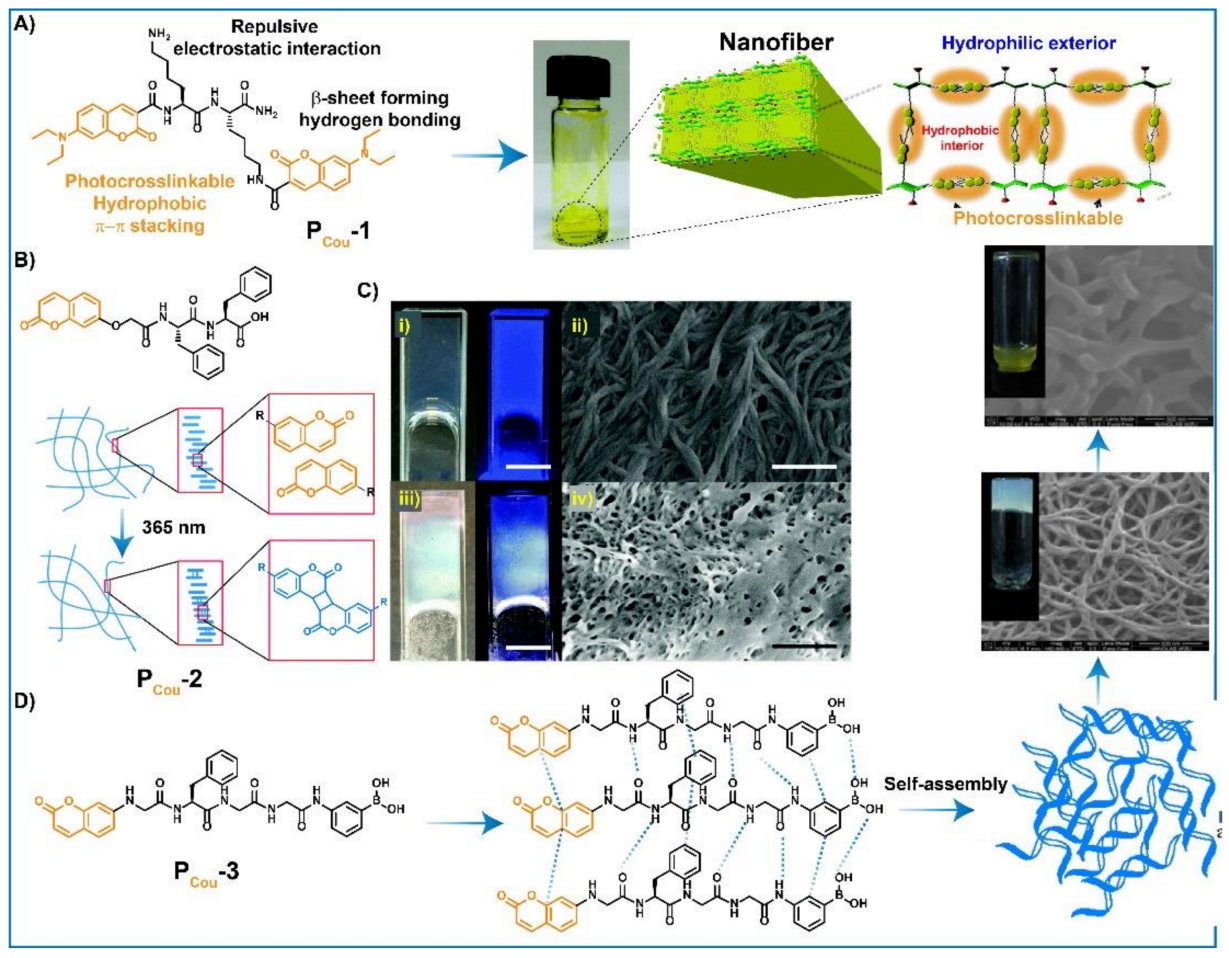
(**A**) Molecular structure of a coumarin-based hydrogelator followed by light-induced self-assembly to form a gel with nanofibrous structure. Adapted from Ref. [122] Copyright 2015 The Royal Society of Chemistry. (**B**) Chemical structure and photo-induced dimerization of a coumarin-peptide conjugate; (**C**) Photographs of gel: (i) under normal and UV light, (ii) Microscopic image displays the fibrous network, (iii) Photographs of dimerized gel under normal and UV light, (iv) Microscopic image of photo-induced dimerized gel. Adapted from Ref. [22] Copyright 2015 The Royal Society of Chemistry. (**D**) Chemical structure and plausible self-assembly of a coumarin-peptide conjugate and the light-sensitiveness. Adapted from Ref. [123] Copyright 2019 Elsevier.

As a proof-of-principle, in 2015, Parquette and Grinstaff et al. reported an LMWG (P_Cou_−1) in which two coumarin moieties are connected to both the N-terminal and N-ε side chain free amine (-NH_2_) of a well-explored *β*-sheet forming dipeptide, dilysines (Figure 8A) [122]. The gelator undergoes self-assembly in pure water, saline, and PBS to form a bright yellow-colored gel which collapses to an insoluble precipitate upon prolonged irradiation (>7 days) at 365 nm because of the enhanced dimerization between coumarin units (Figure 8A). As a consequence, the storage modulus (G′= ~150 Pa) was enhanced compared with the original gel (G′= ~20 Pa), indicating UV-light induced enhanced stiffness in dimerized gel (Table 1). Inspired by this, Adam’s group reported a self-supporting, transparent gel made of a popular Phe-Phe dipeptide motif and N-terminal protected coumarin unit in the same year (P_Cou_−2, Figure 8B) [22]. Under UV light, the gel fluoresces blue light. Interestingly, the gel only experiences turbidity followed by opacity when exposed to UV light irradiation (Figure 8C). Interestingly, light irradiation (15 min) enhanced both storage (G′) and loss (G″) modulus in comparison to the primary gel (Table 1). The amplified moduli value can be ascribed to both the photodimerization (covalent bonding) and dimerization (non-covalent bond) of the coumarin moieties in the gelator. In 2019, Wu and Gao et al. demonstrated a photocleavable LMWG based on 7-amino coumarin (P_Cou_−3, Figure 8D) [123]. The gelator forms an opaque gel through π−π stacking between Phe units and coumarin. Interestingly, the gel exhibits a spiral-shaped three-dimensional fibrous network formed via intermolecular H-bonding (Figure 8D). The gel undergoes gel to yellow colored sol when irradiated with 365 nm wavelength light. Surprisingly, the gel is stable at 420 nm and 630 nm light. Another exciting feature of this gel is the photocleavage property of the C-N bond in the 7-amino position of coumarin, which makes the gelator from the conventional dimerized coumarin gels.

**Figure 9:**
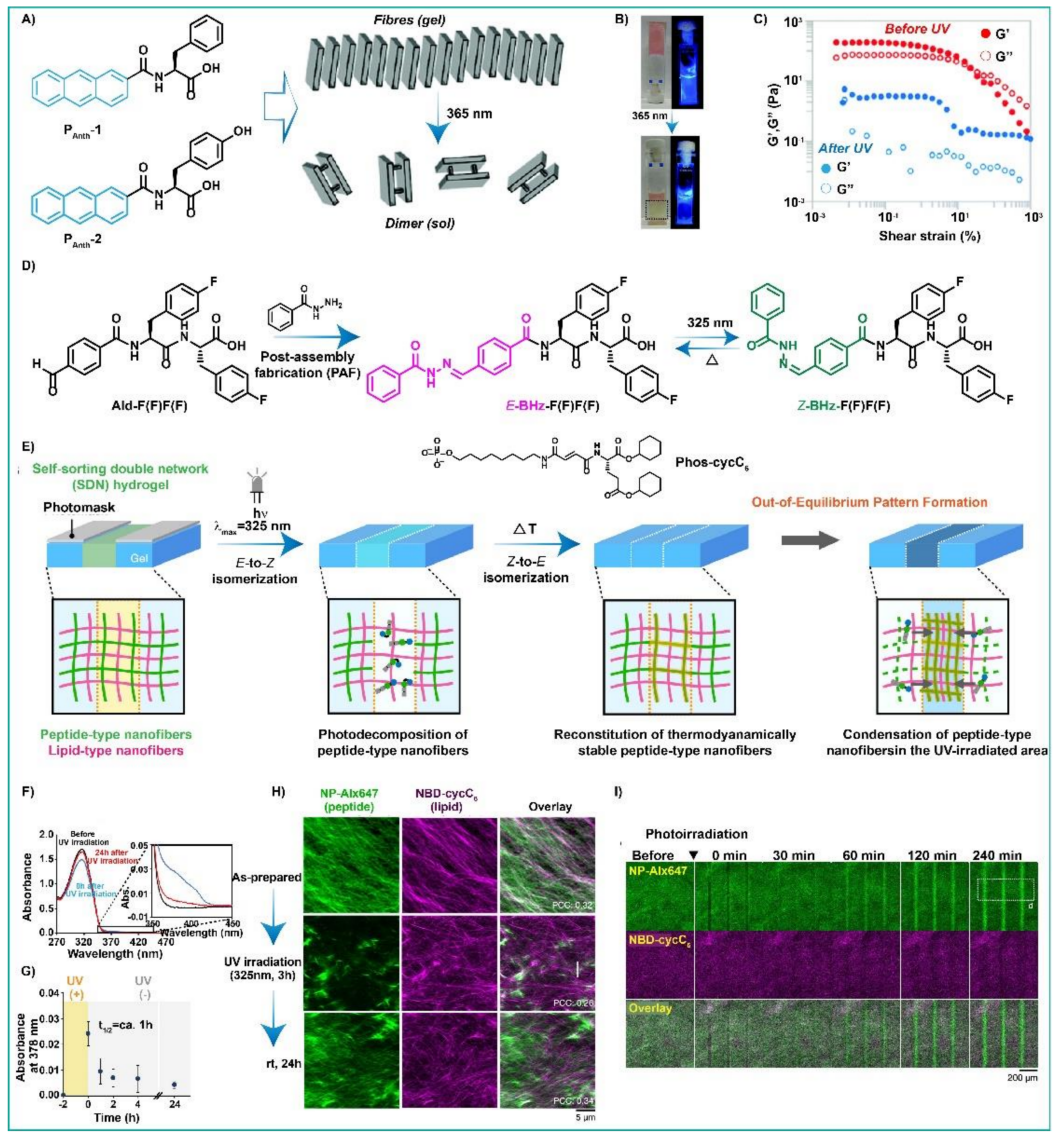
(**A**) Chemical structure and schematic light-induced dimerization of anthracene-based peptide gelators (P_Anth_−1 and P_Anth_−2); (**B**) Photographs of gels before and after UV-light irradiation, the irradiated zone is marked with a dotted line; (**C**) Rheological analysis of P_Anth_−1 gel before and after UV-light irradiation. Adapted from Ref. [102]. Copyright 2020 The Royal Society of Chemistry is not applicable here as this Ref. is licensed under a Creative Commons Attribution 3.0 Unported Licence. (**D**) Molecular structures of the peptide-type gelator (Ald-F(F)F(F)/BHz-F(F)F(F)) and lipid-type gelator (Phos-cycC_6_); Redrawn from Ref. [92]; (**E**) Schematic illustration of the phototriggered out-of-equilibrium pattern formation utilizing peptide-type nanofibers in the SDN hydrogel network; (**F**) UV-Vis spectra of the above-mentioned schematic (**E**); (**G**) Time course UV data at 378 nm before and after photoirradiation; (**H**) High-resolution Airyscan CLSM images of the SDN hydrogels; (**I**) Time-lapse CLSM images of the BHz-F(F)F(F)/Phos-cycC_6_ hydrogel before and after photoirradiation using a photomask. Adapted from Ref. [92] Copyright 2021 American Chemical Society.

In a similar vein, anthracene also undergoes a light-triggered [4 + 4] photodimerization mechanism in which the short-life excimer of photoexcited diene undergoes a transition into the cyclooctane structure (Figure 1E) [104,157,158,159]. Additionally, the self-assembly nature of anthracene through hydrophobic and π-π interactions inspires the researchers to create anthracene-based hydrogelators [102,160,161,162]. For example, Adam’s group demonstrated anthracene dipep-tidedipeptide-based co-assembled hydrogel [163]. Later, Das et al. reported 9-anthracenemethoxycarbonyl (Amoc)- protected dipeptides consisting of PheLeu, PheTyr, and PhePhe that undergo self-assembly under physiological conditions (pH 7.4, 37 °C) to smart, robust hydrogels with injectable and self-healable characteristics [160,161]. In recent years, although there have been few examples of anthracene-linked peptide hydrogels, light-induced self-assembly is very limited. In 2020, Webb et al. reported modified amino acid to prepare light-responsive hydrogel (Figure 9A) [102]. To achieve that N-terminal of the amino acid (here Phenylalanine, tyrosine) was protected with anthracene moieties, and undergoes self-assembly to form a supramolecular self-supporting transparent gel in the presence of different triggers such as glucono-δ-lactone (GdL), a range of salts (NH_4_ +, Na+, K+, GlcN·HCl and GlyNH_2_·HCl), cell culture media and heating-cooling process (Figure 9A) [102]. The pH of the resultant gel was found to be around pH 11, and to achieve the physiologically relevant pH, glycinamide (GlyNH_2_·HCl, trigger A) and glucosamine (GlcN HCl) were added to the mixture. Rheological analysis revealed that the metal-induced gel exhibited elastic modulus around 70 Pa, whereas B-(cell-culture media) and C- (GlcN HCl in cell-culture media) triggered gel showed around 2000 Pa, implying more stiffness (Figure 9B, Table 1). To check its light sensitivity, the C-triggered formed hydrogel in the cuvette was irradiated with 365 nm LED, and eventually, the irradiated regions appeared yellow with decreased emission intensity. The gel to sol transition appeared only in irradiated areas after ca. 15 min. In contrast, the other areas remain in gel form even after 1 h of irradiation, indicating chemical changes induced disassembly. The dimerization was confirmed with the help of NMR spectroscopy and mass spectrometric techniques. Finally, the light-induced property was employed to release encapsulated cells for standard biochemical analysis.

Benzoylhydrazone is another interesting moiety that also exhibits reversible *E*-*Z* isomerization on photoirradiation, but this moiety is less explored in the context of peptide-based gelators (Figure 1C) [92]. Recently, the group of Itaru Hamachi demonstrated a benzoylhydrazone-based photoresponsive peptide-based self-sorting supramolecular double network (SDN) hydrogel system capable of showing photo triggered out of equilibrium patterns generation (Figure 9D–I) [92]. They exploited the previously developed SDN hydrogel composed of orthogonally self-assembled benzaldehyde-tethered peptide-type gelator (Ald-F(F)F(F)) and a lipid-type gelator (Phos-cycC_6_) as a template to install photo responsive module onto the aldehyde terminal of the peptide using a post assembly fabrication (PAF) approach [164] (Figure 9D) without disturbing the SDN network structure. This newly generated benzoylhydrazone moiety of the peptide [BHz-F(F)F(F)] in the SDN hydrogel can undergo *E*-*Z* isomerization under UV light illumination, causing a perturbation in the packing mode resulting in destabilization of the fibrous network. On thermal agitation, the destabilized network could again get re-stabilized due to thermal Z-E isomerization, and this fiber destruction and reconstruction were assessed by Confocal Laser Scanning Microscopic (CLSM) images (Figure 9H). Moreover, as the other network of the SDN hydrogel is composed of lipid-type gelator (Phos-cycC_6_), it could not show such light responsiveness. Thus lipid-type gelator fibers remain intact (Figure 9E,H). At this point, it is worth mentioning that before applying their hypothesis of photo and light-induced changes in the developed SDN system, they separately synthesized [BHz-F(F)F(F)] using the PAF approach and tested its photo-thermo responsiveness (Figure 9D,F,G). Following the selective photoresponsive behavior of the benzoylhydrazone-containing network of the SDN hydrogel, when photoirradiation is conducted using a photomask, the peptide-type nanofibers are selectively destroyed in the limited exposed area, and subsequent incubation under darkness causes the nanofibers to reconstruct in the same area. Furthermore, additional thermal incubation causes spatial condensation of [BHz-F(F)F(F)] nanofibers in the photoirradiated areas and concurrent nanofiber depletion in the nonirradiated areas (Figure 9I). Finally, they fabricated unique complex patterns, namely (1) two-line patterns from a one-line photomask and (2) grid-like patterns from a one-line photomask by photomasks using their developed photo/diffusion-coupled out-of-equilibrium approach. 

**Figure 10:**
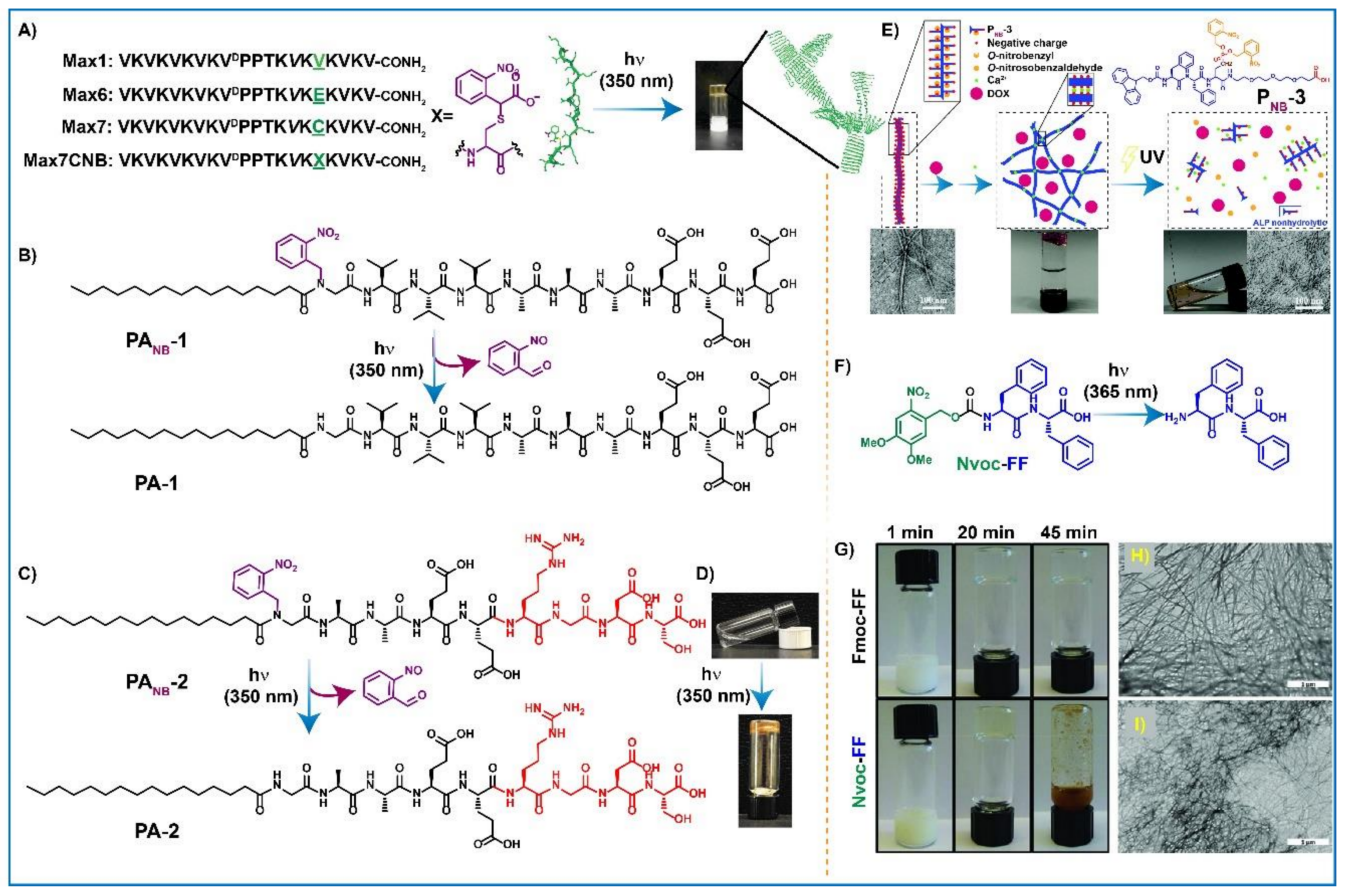
(**A**) Molecular structure of photo-sensitive peptides; Adapted from Ref. [124] Copyright 2005 American Chemical Society. (**B, C**) Light-induced photocleavable peptide amphiphiles; (**D**) Light-assisted sol-gel transition of photocleavable PA. Adapted from Ref. [126] Copyright 2009 John Wiley and Sons. (**E**) Plausible self-assembly behavior of nitro benzyl-conjugated peptide. Adapted from Ref. [127] Copyright 2020 Royal Society of Chemistry. (**F**) Chemical structure of Nvoc capped peptide and its photo-induced behavior; (**G**–**I**) Gel photographs and respective microscopic images. Ref. [128] Copyright 2018 John Wiley and Sons.

In the same context, 2-nitrobenzyl and 6-nitroveratryloxycarbonyl (Nvoc) photocleavable groups were reported by Pochan and Schneider [124], Stupp [126], Shabat and Adler-Abramovich’s group [128], who created a pathway to understand and design light responsive self-assembly/disassembly. In a pioneering work by Schneider, the unfolded gelator (MAX7CNB) transforms into a *β*-hairpin folded conformation followed by efficient self-assembly (both facial and lateral) to form a transparent gel when exposed to UV irradiation (λ > 300 nm) (Figure 10A) [124]. In this process, the rheological moduli of decaged hydrogel (G′ (~10^3^ Pa), G″ (~10^2^ Pa)) were found to be lower in comparison to the original one (G′ (10^3^–10^4^ Pa), G″ (~10^2^ Pa), Table 1). Later, Stupp and co-workers explored the self-assembly/disassembly process of 2-nitrobenzyl group appended peptide amphiphiles (PA_NB_−1, Figure 10B) [125]. In response to UV light (350 nm), the quadruple helical fibers transformed into cylindrical fibrils. Based on this work, the same group engineered another 2-nitrobenzyl conjugated PA (PA_NB_−2, Figure 10C), which experiences a sol-to-gel transition in the presence of light (Figure 10D) [126]. Intriguingly, under the self-assembly condition, PA remains as a solution, but it forms a gel in the presence of charge-screening Ca^2+^ salts when triggered with light. The sol nature of the PA is because of the combined effect of the bulkiness of the photo caging 2-nitrobenzyl group and the use of a weaker *β*-sheet-forming motif in comparison to the previously reported sequence ((GA_2_E_2_) vs. (GV_3_A_3_E_3_)). The storage modulus (G′ = ~10^5^) was found to be higher than the loss modulus (G″ = ~10^4^), indicating the gel behavior and which is comparable to previously reported PA_NB_−2 (Table 1). Considering all the above-mentioned references into consideration, in 2020, Chen et al. demonstrated an advanced LMWG (P_NB_−3), which undergoes sol-gel-sol transition under the influence of sequential metal and light induction (Figure 10E) [127]. The peptide is composed of a well-established Phe-Phe dipeptide motif, o-nitrobenzyl protected phosphonated serine, and a short PEG chain with a C-terminal-free carboxylic acid. The classical Fmoc moiety protects the N-terminal of the sequence. The gelator forms soluble fibers in pure water (pH 7.4). However, the peptides solution (1.7 mM) transforms into a transparent gel in the presence of Ca^2+^ ions (1.7–3.4 mM). This can be attributed to the peptide crosslinking through coordination interaction between carboxylate anions and divalent metal ions. Under UV-light irradiation (365 nm), the protected group on phosphonate gets removed, resulting in the decaging of the negative charges. Therefore, the gel again is dissolved. The rheological analysis confirmed the gel character (G′ > G″, Table 1), and interestingly, the moduli value belongs to the soft peptide hydrogel category, indispensable for drug delivery applications. Inspired by the self-assembly of Fmoc-FF, Adler-Abramovich and Shabat et al. reported Nvoc protected LMWG, Nvoc-FF, which endures self-assembly in water to form 3D stable, self-supporting, transparent hydrogel [128]. In response to light, the hydrophobic aromatic Nvoc group gets cleaved, and thus, the gels completely degrade (Figure 10F,G). The higher value of G′ compared with G″ confirmed gel properties. Under UV-light irradiation, the G′ value continuously decreased because of the gel decomposition, and after some time, the gel liquified.

## 3. Challenges, Future Prospective, and Conclusions

Over the past decades, stimuli-responsive peptide gelators have taken the spotlight as powerful building blocks to fabricate numerous molecular and biomolecular systems and smart materials using supramolecular self-assembly strategies in both aqueous and organic media. Among the stimulus, light has received considerable attention in developing peptide-based photo-responsive systems and materials. Unlike pH, heat, ionic strength, etc., peptides are not usually responsive toward light; thus in order to make them photoresponsive, linking with light-sensitive chromophoric units is necessary. The light-sensitive unit, on illumination with light having a specific wavelength, undergoes photoreactions such as molecular switching (*E*-*Z*/*Z*-*E* isomerization), bond rupture, or bond formation leading to a change in physical and chemical properties like dipole moment, conjugation, geometric structures, and electronic properties, etc. These physicochemical changes dictate the modified peptide to undergo assembly/disassembly or any other phase transition, thus making the peptides responsive toward the light. Hence, in order to achieve the photoresponsiveness in the peptide, proper selection of photoresponsive units is crucial not only from the point of system development but also for desired properties and functions to achieve. Indeed, the cross-fertilization of peptide chemistry with photo-chemistry not only provides biocompatibility to the hybrid materials (gel) but also facilitates the structural control of peptide assemblies at the microscopic and macroscopic levels using light. In this short review, we have highlighted the recent developments made in the field of photo-sensitive peptide gelators, where a variety of photo-sensitive units are integrated with peptides either covalently or non-covalently. Many recent developments in this direction allow us to think about new possibilities and opportunities in advanced systems development and applications such as controlled drug delivery, modulation of cell behavior, development of adaptive and self-healing materials, catalysis, etc. Although a substantial effort has been devoted to designing and developing light-responsive peptide gelators with varying peptide sequences and molecular photo-switches, however, the present systems are restricted to proof-of-concept studies in the context of applications, particularly in the area of biomedical applications, and the clinical trial seems to be a bit away. One of the most common challenges is that majority of the light-responsive units need UV light to respond. This UV-irradiation can damage tissue, making this system imperfect for cellular applications. Moreover, UV light has poor penetration depth into human cells. To overcome these challenges, new photo-responsive units must be designed and developed to respond to low-energy light such as NIR light and to perform on-demand tasks. However, the use of low-energy light can slow down the drug release kinetics. Additionally, the heat generated by light irradiation can also cause cell mortality, which should also be considered while designing photo-responsive peptide gelators for biomedical applications. On the other side, the photoswitches are organic molecules. Due to their poor solubility in an aqueous medium, the majority of the systems are developed either in pure organic solvents or in the mixtures of organic-aqueous milieu, thus limiting in vitro and in vivo biological applications. Maturity in design principal and evaluation of biotoxicity, biostability, bioavailability, and drug release kinetics of the developed system can improve the biological applications step by step. Most of the developed photo-sensitive peptide gelators comprise only one type of photo-switch to respond to a specific wavelength. However, peptide gelators having two or multiple different photo-switch that can smartly respond to several wavelengths is still to be developed to achieve selective and precise control over multifunctional responsiveness or orthogonal photo-modulation, which can give access to two or more properties in a single peptide gel system. Currently, supramolecular assembly is moving towards the non-equilibrium approach where ‘chemical fuels’ are being exploited to device adaptive systems with multiple interactions, complex structures, and functions with spatio-temporal control, in short, ‘life-like’ systems [165,166]. In this context, light as a fuel could be a better alternative of ‘chemical fuel’ to device ‘life-like’ autonomous functional systems because of its special resolution, lack of waste production, and wavelength selectivity. In fact, a few ‘light fueled’ dynamic and autonomous peptide assemblies and gels are reported, which are limited to the system development only [121,167]. Development of transient and self-abolishing color and ink, a temporary memory device, transient electric circuit, signal transduction and catalysis, pre-programmed loading and release of pharmaceuticals, etc., could be future applications of such dynamic light responsive (fueled) peptide gels. We believe that with the proper synthetic toolbox, light responsive peptide gelators could create a new avenue to achieve next-generation advanced systems with emergent behaviors and to mimic properties and behaviors of living systems.

Although many challenges need to be addressed, we believe that the rational design of photoactive units and peptides, better understanding of gelation mechanism and photoswitching kinetics, and introduction of a non-equilibrium approach can collectively offer new generation peptide-based photo-responsive materials for practical applications. Therefore, this field still demands a good amount of advanced research.
